# Weight loss dynamics during combined fluoxetine and olanzapine treatment

**DOI:** 10.1186/1471-2210-4-27

**Published:** 2004-10-21

**Authors:** Jennifer A Perrone, Janet M Chabla, Brian H Hallas, Judith M Horowitz, German Torres

**Affiliations:** 1 Department of Neuroscience, New York College of Osteopathic Medicine of New York Institute of Technology, Old Westbury New York, 11568 USA; 2 Department of Psychology, Medaille College, Buffalo New York, 14214 USA

## Abstract

**Background:**

Fluoxetine and olanzapine combination therapy is rapidly becoming an effective strategy for managing symptoms of treatment-resistant depression. Determining drug-drug interactions, drug metabolism and pharmacokinetics is of particular interest for revealing potential liabilities associated with drug augmentation in special patient populations. In the current studies, we chronically administered fluoxetine and olanzapine in non-stressed rats to extend our previous findings regarding body weight dynamics.

**Results:**

Chronic fluoxetine (10 mg/kg) and olanzapine (5 mg/kg and 0.5 mg/kg) treatment decreased weight gain irrespective of olanzapine dosing. At the 10 mg/kg and 5 mg/kg dose, respectively, fluoxetine and olanzapine also significantly reduced food and water consumption. This pharmacodynamic event-related effect, however, was not observed at the 10 mg/kg and 0.5 mg/kg dosing paradigm suggesting differences in tolerability rates as a function of olanzapine dose. The decrease in weight gain was not associated with apparent changes in glucose metabolism as vehicle- and drug-treated rats showed undistinguishable serum glucose levels. The combination of fluoxetine and olanzapine in rats yielded drug plasma concentrations that fell within an expected therapeutic range for these drugs in psychiatric patients.

**Conclusions:**

These data suggest that fluoxetine and olanzapine treatment decreases weight gain in rats; a pharmacodynamic event-related effect that differs considerably from what is observed in the clinical condition. The possibility of mismatched models regarding body weight changes during drug augmentation therapy should be seriously considered.

## Background

Treatment-resistant depression is a serious issue in psychiatry as a significant number of affected individuals show an inadequate response to single antidepressant therapy. An emerging strategy to achieve maximum mood stabilization for treatment-resistant depression, bipolar illness and depression with psychotic features is the augmentation of fluoxetine (Prozac) with novel anti-psychotic agents such as olanzapine (Zyprexa). Indeed, a number of clinical trials have suggested that such an augmentation strategy offers superior efficacy for treating resistant major depression when compared with either fluoxetine or olanzapine alone [1-3]. Despite the apparent clinical benefits of this drug strategy, little is known about the mechanisms by which fluoxetine plus olanzapine actually function to relieve depression. The limited literature on this issue suggests that drug augmentation therapy, at least in the rat brain, is likely to be more complicated and perhaps more indirect than a simplistic version of fluoxetine or olanzapine would imply [4-7]. For instance, whereas fluoxetine and olanzapine alone activate several signaling pathways involved in cell survival and plasticity [8-10], drug augmentation therapy reduces the levels of certain transcription factors (e.g., cAMP response element binding protein and FOS-like proteins) implicated in the chemical circuitry (e.g., prefrontal cortex and hippocampus) underlying emotional behaviors [5]. Consequently, it is conceivable that fluoxeine plus olanzapine treatment is effective against treatment-resistant depression due to their combined actions on numerous brain regions and various interconnected intracellular signaling pathways that ultimately promote some type of prophylactic effect.

We have previously shown that sub-chronic (i.e., 7 days) administration of fluoxetine plus olanzapine results in a significant reduction of weight gain in rats [5]. This finding is of significant interest as fluoxetine and olanzapine alone have distinct and opposite effects on body weight dynamics in both rodents and humans. For example fluoxetine often reduces food intake and thus body weight in rats during sub-chronic and chronic (i.e., 21 days) drug regimens [11], an effect apparently mediated by fluoxetine impact on serotonin (5-HT) signaling pathways [12]. In sharp contrast, treatment with olanzapine is associated with significant weight gain in schizophrenic patients, a serious side effect that may increase the risk for type II diabetes and may also lead to treatment non-compliance [13,14]. In this case it is thought that olanzapine's particular affinity for 5-HT (5-HT_2A_), dopamine (DA; D_2–4_), acetylcholine muscarinic (ACh; M_1_–M_5_) and histamine (H_1_) receptors distributed widely in limbic neural circuits may somehow account for the pharmacological basis of olanzapine-induced weight gain [15]. Needless to say, understanding body weight dynamics in relation to drug augmentation therapy is of critical importance if we are going to gain further knowledge on the mechanisms of therapeutic action and side effect profile of anti-depressant medications. In this regard, appetite disturbances are noted in many medicated depressed patients and several peptide transmitters implicated in feeding behavior co-exist in the hypothalamus and may therefore be involved in the onset of affective states [16]. In this study, we have examined in more detail the effects of fluoxetine (10 mg/kg) plus olanzapine treatment on rat body weight during the time course of 18 days under two olanzapine doses: 5 and 0.5 mg/kg. In addition, we have measured blood levels of these two drugs using gas-chromatography-mass spectrometry (GC-MS) to assess their combined pharmacology and their correlation to body weight dynamics.

## Results

**Figure 1:**
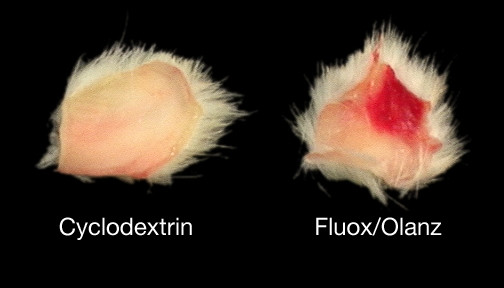
**Tissue necrosis during chronic fluoxetine (fluox) and olanzapine (olanz) treatment. **This figure depicts equally excised peritoneal cavities of males injected IP with either a vehicle-solution (cyclodextrin) or the above drug combination pattern for 18 consecutive days. Note the extent of tissue damage (~1 cm wide) at the site of drug administration. Focal necrosis was evident in drug-treated rats irrespective of olanzapine dosing.

All rats tolerated the fluoxetine plus olanzapine regimen well. There were no mortalities as a result of 18 days of drug administration in any of the rat groups tested. The only apparent untoward side effect was tissue necrosis in the peritoneum of rats injected with fluoxetine plus 5 or 0.5 mg/kg olanzapine (Fig. 1). Thus, fluoxetine appears to produce focal *necrotising vasculitis *within the site of injection. The necrotic properties of the above antidepressant have previously been reported [17]. Olanzapine, on the other hand, does not produce tissue necrosis in the peritoneal cavities of rats when administered alone (data not shown).

**Figure 2:**
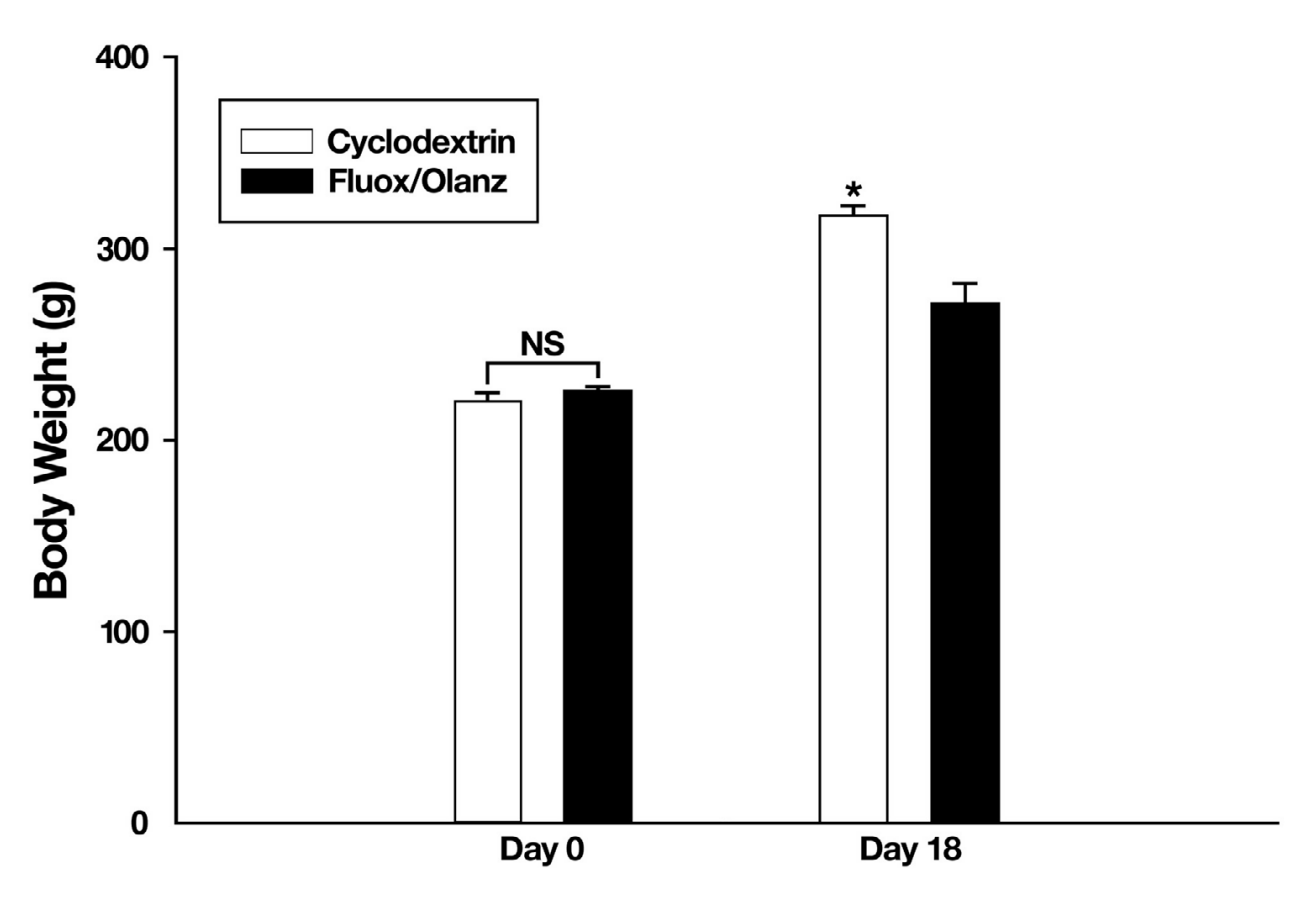
**Body weight changes during chronic fluoxetine (fluox, 10 mg/kg) and olanzapine (olanz, 5 mg/kg) treatment. **Rat body weights were recorded before and after drug augmentation therapy. Data represent means ± SEM. N = 5–7 animals per group. *P ≤ 0.05 when compared with drug-treated rats. NS = not significant.

**Figure 3:**
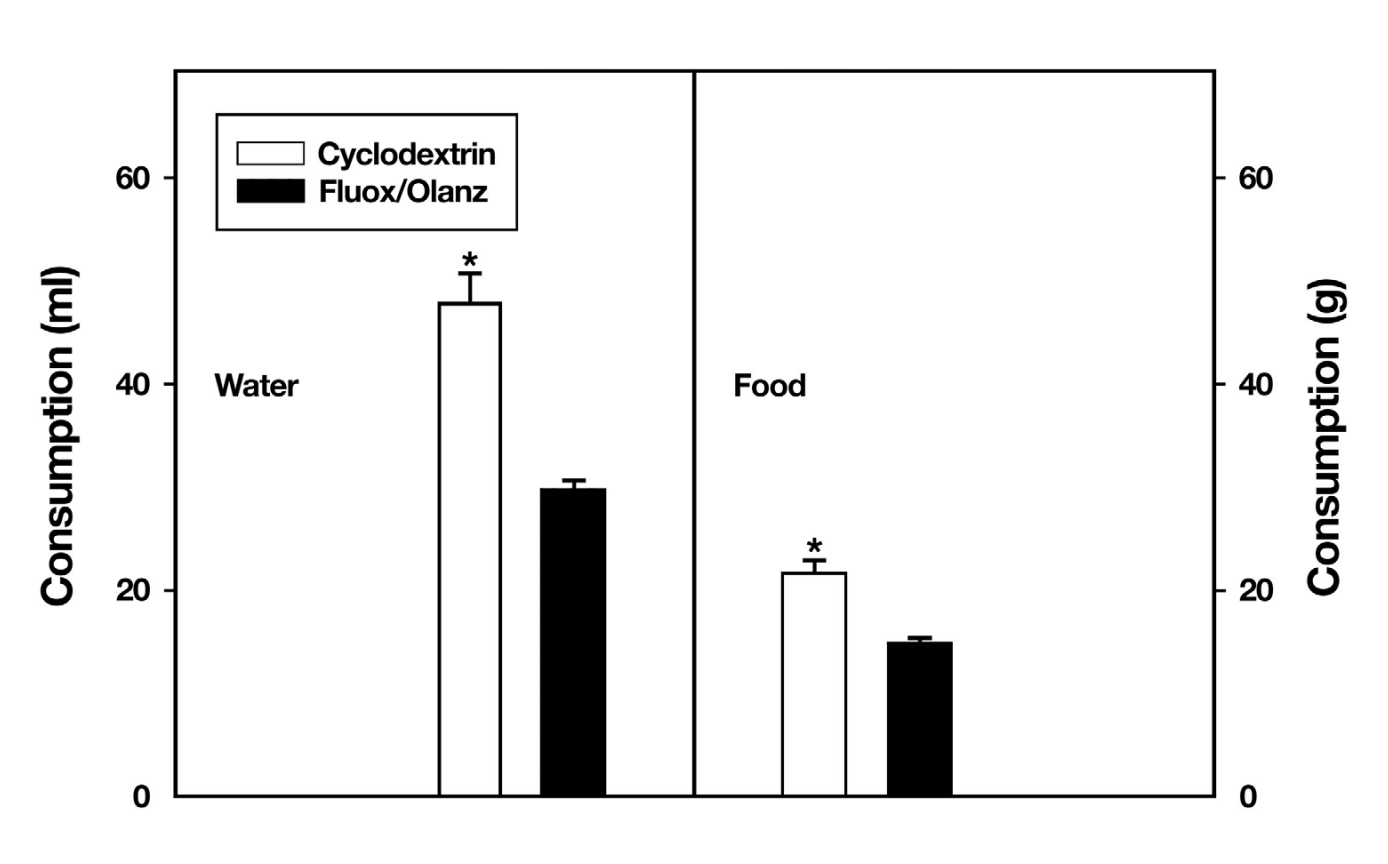
**Changes in food and water intake during chronic fluoxetine (fluox, 10 mg/kg) and olanzapine (olanz, 5 mg/kg) treatment. **Rats under this combined drug regimen showed a significant reduction in the consumption of nutrients and fluids at day 10 and 12 of drug therapy, respectively. Data represent means ± SEM. N = 5–7 animals per group. *P ≤ 0.05 when compared with drug-treated rats.

All rats showed a steady increase in body weight during the 18 days of cyclodextrin or fluoxetine plus olanzapine treatment. However, fluoxetine in combination with olanzapine significantly retarded this growth rate (P ≤ 0.001) when compared with the vehicle-treated group (Fig. 2). This weight loss, beginning on day 7 of treatment, was observed equally in both the 5 and 0.5 mg/kg olanzapine-treated groups. At this time, rats administered with cyclodextrin showed body weights of 275.6 ± 3.7 g, whereas fluoxetine plus 5 mg/kg olanzapine-treated animals showed weights of 248.1 ± 4.4 g (at the 95% confidence interval for differences between means: 13.8 to 41.2, P ≤ 0.001). Along the same lines on day 7, rats treated with fluoxetine plus 0.5 mg/kg olanzapine showed body weights of 254.1 ± 3.7 g. In contrast, their vehicle-treated cohorts showed weights of 277.8 ± 7.3 g (at the 95% confidence interval for differences between means: 6.90 to 40.41, P ≤ 0.01). The magnitude of this difference in body weight increased further on day 14 and was firmly established by day 18 of drug augmentation therapy (Fig. 3). Thus, fluoxetine treatment, irrespective of olanzapine's ability to cause weight gain, produces a gradual and considerable weight loss in male rats. In general, these findings are consistent with mono-therapy studies where chronic olanzapine treatment invariably leads to weight loss in rodents [18,19].

**Figure 4:**
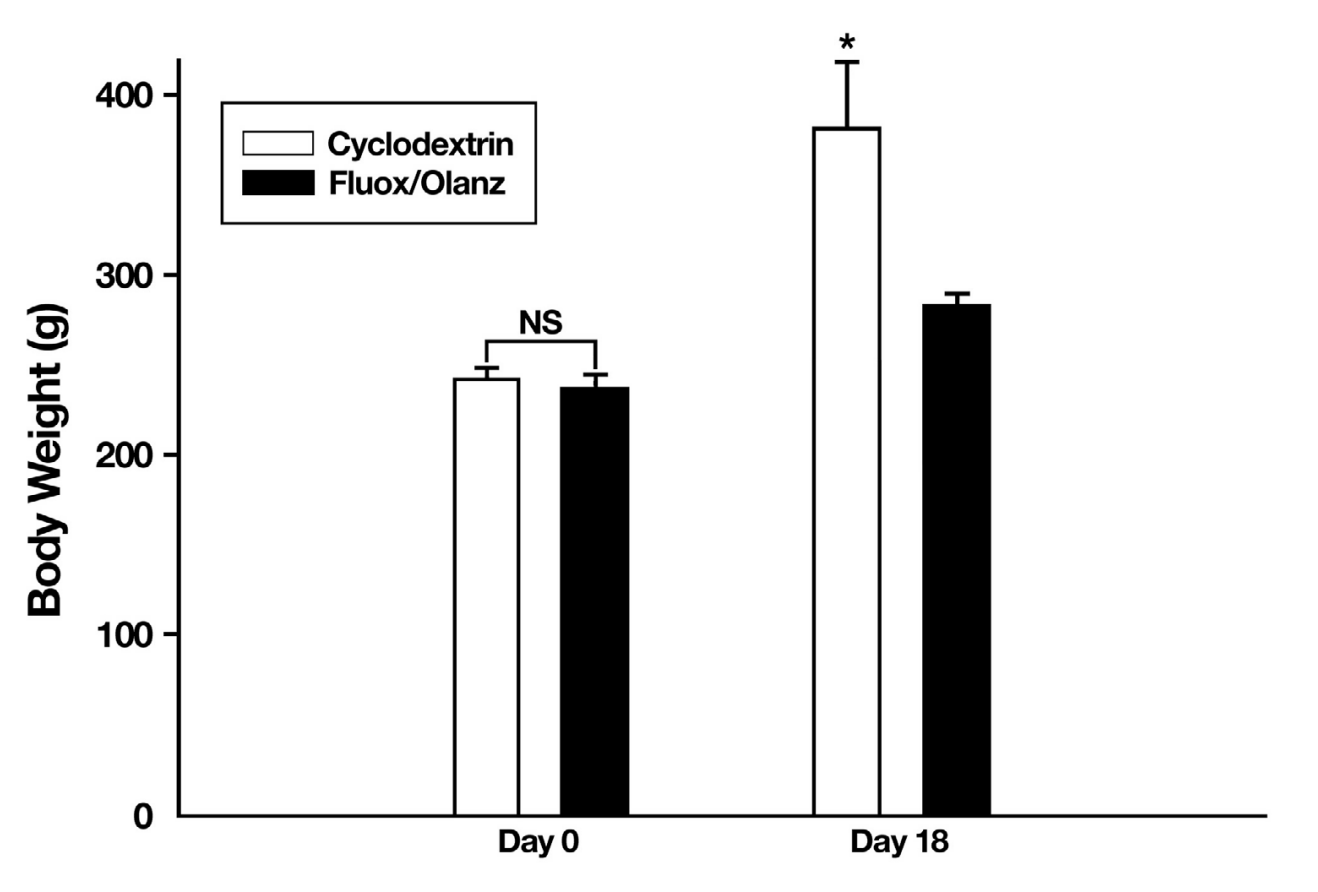
**Body weight changes during chronic fluoxetine (fluox, 10 mg/kg) and olanzapine (olanz, 0.5 mg/kg) treatment**. Rat body weights were recorded before and after drug augmentation therapy. Data represent means ± SEM. N = 5–7 animals per group. *P ≤ 0.05 when compared with drug-treated rats. NS = not significant.

The fact that fluoxetine plus olanzapine treatment for 18 days retards the continuous weight gain observed in cyclodextrin-exposed rats suggests at least two testable possibilities. First, rats exposed to drug augmentation therapy might be eating less than their cyclodextrin-treated cohorts. Second, administration of fluoxetine plus olanzapine might be altering glucose metabolism of drug-treated animals. To test the first possibility, we measured average food intake over a 12 hr period of the dark cycle in rats treated with fluoxetine plus 5 mg/kg olanzapine. On day 10 of drug treatment, rats exposed to this drug augmentation regimen ate significantly less (t_10 _= 5.5, P ≤ 0.001) than vehicle-treated animals (Fig. 4). Interestingly, the same group of rats also showed a significant reduction in water intake (t_10 _= 6.7, P ≤ 0.01) when compared with cyclodextrin-treated animals (Fig. 4). Thus, rats exposed to fluoxetine plus 5 mg/kg olanzapine are eating (~32%) and drinking (~38%) less at day 10 and 12 of drug treatment, respectively.

**Figure 5:**
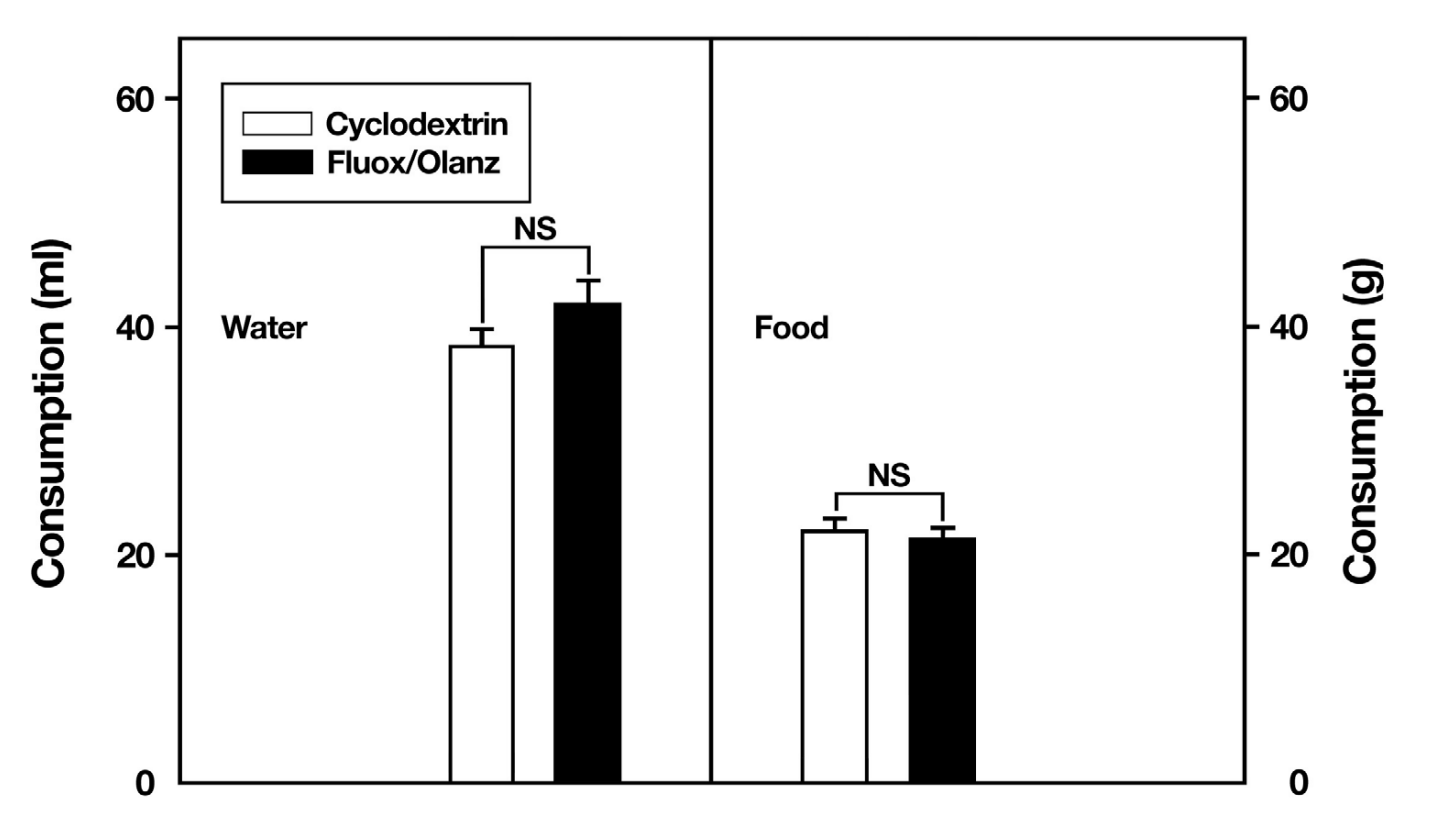
**No changes in food consumption or water intake during chronic fluoxetine (fluox, 10 mg/kg) and olanzapine (olanz, 0.5 mg/kg) treatment. **Rats under this combined drug regimen did not show an apparent reduction in the consumption of nutrients and fluids at day 10 and 12 of drug therapy, respectively. Data represent means ± SEM. N = 5–7 animals per group. NS = not significant.

**Figure 6:**
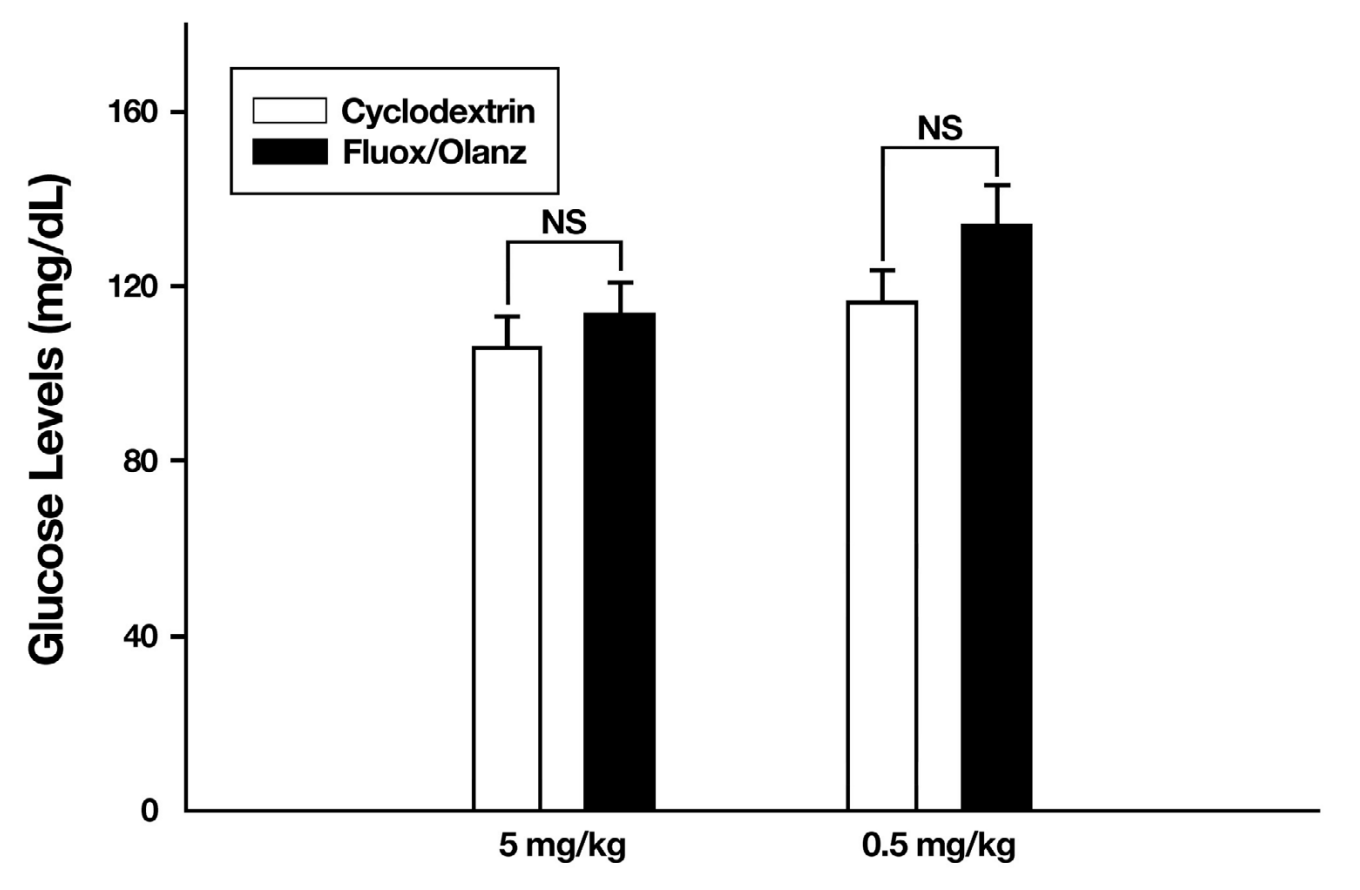
**No changes in fasting glucose levels after chronic fluoxetine (fluox, 10 mg/kg) and olanzapine (olanz, 5 mg/g or 0.5 mg/kg) dosing. **Rats under the depicted drug regimens did not show overt differences in glucose metabolism. Data represent means ± SEM. N = 5–7 animals per group. NS = not significant. Non-fasting glucose levels in rats are typically in the range of 155–242 mg/dL.

In contrast to the above findings, animals injected first with fluoxetine and then 15 min later with 0.5 mg/kg olanzapine did not show differences in food (t_10 _= 2.2, P ≥ 0.05) or water (t_10 _= -1.2, P ≥ 0.05) consumption during the dark cycle when compared with cyclodextrin-treated rats (Fig. 5). Thus, although fluoxetine plus 0.5 mg/kg olanzapine-treated rats show a significant and progressive weight loss at days 7, 14 and 18, this weight loss is not associated with reductions in food or water consumption. This finding suggests that chronic olanzapine treatment is apparently modifying feeding behavior in rats, an effect particularly conspicuous at the 5 mg/kg dose. To test the second possibility, that chronic fluoxetine plus olanzapine is altering the metabolism of drug-treated animals, we measured their blood glucose levels under fasting conditions (Fig. 6). Glucose levels at the time of sacrifice were not significantly different between cyclodextrin- and drug-treated animals at either the 5 mg/kg olanzapine dose (t_10 _= -0.73, P ≥ 0.05) or the lower 0.5 mg/kg olanzapine dose (t_10 _= -1.4, P ≥ 0.05). Thus, changes in glucose metabolism are not the proximate cause affecting the differential body weight dynamics, nor the differential consumption of food and water among vehicle- and drug-treated rats. Along the same lines, levels of the hormone leptin did not differ between cyclodextrin- and drug-treated animals (data not shown), thus suggesting that chronic fluoxetine and olanzapine drug therapy does not affect leptin messages under fasted conditions in male rats.

We next assessed the pharmacokinetic profile of fluoxetine and olanzapine in rats treated with the above drug combination pattern (Table 1). At doses of 10 mg/kg and 5 mg/kg respectively, fluoxetine plasma levels ranged from 62 ng/mL to 476 ng/mL. In contrast norfluoxetine levels ranged from 292 ng/mL to 1175 ng/mL. Norfluoxetine is the only identified active metabolite of fluoxetine; it is formed through N-demethylation of the parent molecule. Plasma concentrations of olanzapine ranged from 74 ng/mL to 301 ng/mL. At doses of 10 mg/kg fluoxetine and 0.5 mg/kg olanzapine, levels of the antidepressant drug ranged from 276 ng/mL to 576 ng/mL, whereas those for norfluoxetine ranged from 266 ng/mL to 966 ng/mL. Olanzapine levels at this low dose were in the range of 34 ng/mL and 65 ng/mL. When inter-group comparisons of drug plasma concentrations were made between fluoxetine (10 mg/kg) in combination with olanzapine at either the 5 mg/kg or the 0.5 mg/kg dose range, differences in mean values of the two groups did not vary enough (P ≥ 0.05) to reject the possibility of random sampling variability. A similar statistical trend was observed for norfluoxetine levels after 18 consecutive days of drug treatment; there was not a statistically significant difference (P ≥ 0.05) between the two groups. However, differences in the median values between 5 mg/kg and 0.5 mg/kg olanzapine doses were greater than would be expected by chance (P ≤ 0.001). As expected, rats injected IP with cyclodextrin showed no traces of either fluoxetine or olanzapine levels in plasma (≤5 ng/mL). In general, our GC-MS measurements detect pharmacological and relevant levels of both fluoxetine and olanzapine in rats, a finding consistent with previous reports [4].

**Table 1:** Plasma concentrations of fluoxetine, norfluoxetine and olanzapine after 18 consecutive days of drug augmentation therapy.

*Drug Measured*	*Fluoxetine Dose (10 mg/kg)*	*Olanzapine Dose (5 mg/kg)*
Fluoxetine	344.1 ± 54.2 (ng/mL)	
Norfluoxetine	695.4 ± 118.4 (ng/mL)	
Olanzapine		178.5 ± 34 (ng/mL)*

*Drug Measured*	*Fluoxetine Dose (10 mg/kg)*	*Olanzapine Dose (0.5 mg/kg)*

Fluoxetine	410.0 ± 36.6 (ng/mL)	
Norfluoxetine	501.5 ± 114.7 (ng/mL)	
Olanzapine		46.7 ± 4.4 (ng/mL)

The fact that regardless of olanzapine dosing both rat groups lost equal amounts of body weight is indicative that these two phenomena (i.e., relative drug levels and body weight dynamics) may not be causally related. No discernible changes in brain structure or integrity were found, as assessed by Nissl staining and stereological cell counts conducted within the rat hypothalamus (data not shown). Values are means ± SEM. N = 7 per dosing group. * P ≤ 0.01 when compared with appropriate olanzapine dose.

## Discussion

The present study shows that 18 days of concomitant fluoxetine and olanzapine treatment leads to a significant decrease of weight gain in rats. Given that the above drug combination is particularly effective in treatment-resistant depression, our findings are of interest for revealing potential liabilities associated with its therapeutic use. Here, our data suggest a possible pharmacodynamic event-related effect regarding the action of two psychoactive drugs over time. Indeed, there is a well-established relationship between clinically effective drugs, appetite control and weight changes across diverse patient populations [20]. For instance, weight gain appears to be correlated positively with clinical responses to anti-psychotic medication [21,22]. The combination of fluoxetine and olanzapine in our studies produced weight loss irrespective of anti-psychotic drug dosing. That is, fluoxetine at a fixed dose of 10 mg/kg administered concomitantly with either 5 mg/kg or 0.5 mg/kg olanzapine yielded an approximately 20% mean reduction in body weight for both doses. Therefore, body weight changes associated with the above drug combination are more likely due to the effects of olanzapine or its metabolic pathways (see below). Indeed, this hypothesis is further supported by the fact that although rats treated with 5 mg/kg olanzapine were eating and drinking less than animals injected with a smaller dose (i.e., 0.5 mg/kg), body weight outcome was nevertheless similar for all drug-treated rats. In this regard, it is conceivable that the suppressed consumption of food and water observed in animals injected IP with 5 mg/kg olanzapine might have been the result of malaise, or at least the result of aversion to the hedonic aspects of food and water. However this possibility does not explain, in general, the sustained and consistent decrease in weight gain for all rats treated with fluoxetine and olanzapine. Changes in glucose metabolism were also ruled out as a causal role for the reduction in weight gain and food intake as both vehicle-and drug-treated animals showed undistinguishable serum glucose levels during fasting. Further studies of these questions will yield insight into centrally acting peptides and/or peripherally acting thermogenic mechanisms underlying decreases in weight gain in adult rats.

Placing our results in the framework of clinical situations, decreases in rat weight gain as a result of fluoxetine and olanzapine treatment do not mirror the profile occurring across diverse patient populations. There is evidence that long-term fluoxetine plus olanzapine treatment frequently leads to weight gain in individuals with major depressive disorders with and without treatment-resistant depression [23]. Further, high doses of fluoxetine do not appear to counteract the weight gain often induced by atypical anti-psychotics such as olanzapine [24]. The stark disparity between rat and human studies regarding body weight dynamics raises the possibility of mismatched models for revealing certain liabilities associated with fluoxetine and olanzapine therapy in mood disorders. It is conceivable, for instance, that rats might be more sensitive to the anorectic effects of fluoxetine than humans. Fluoxetine is known to produce anorectic effects that often lead to a decrease in weight gain; a phenomenon observed equally at the experimental and clinical level [11,25,26]. Alternatively, olanzapine metabolism may differ significantly in rats as indirectly suggested by previous reports [27,28]. In this context, olanzapine is metabolized to its 10- and 4'-N-glucuronides, with the 10-N-glucuronide being the most abundant metabolite in humans [15,29]. As the pharmacokinetic and pharmacodynamic profile of olanzapine in rats is relatively obscure [27], it is possible that changes in glucuronidation metabolism in rodents may have impacted the ability of the parent drug to influence heterogeneous population of cells associated with body weight dynamics. From these statements, one might conclude that our findings are not clinically significant and perhaps of limited value for additional investigation. Although the animal data indeed do not support the clinical situation, the above findings could harbor important information as to how species-specific differences limit drug-drug interactions or body weigh regulation, lessons that could influence subsequent studies regarding fluoxetine and olanzapine therapy in more defined experimental settings.

In the present study, measurements of fluoxetine, norfluoxetine and olanzapine plasma concentrations were made to assess their pharmacology after 18 days of combined drug exposure. In general, drug plasma levels fell within the expected therapeutic range typically observed in psychiatric patients. For instance, after 30 days of dosing at 40 mg/day, plasma levels of fluoxetine are in the range of 90–300 ng/mL across diverse patient populations [15]. In our animal studies, at a dose of 10 mg/kg (IP), mean plasma concentrations achieved were in the range of 300–400 ng/mL after 18 days of combined drug treatment. Oral doses of olanzapine at 20 mg/day often yield plasma levels of 20–100 ng/mL in healthy volunteers and in patients with schizophrenia [30]. Concentrations ≥80 ng/mL are considered threshold for the occurrence of adverse effects. In our present study, at a dose of 5 mg/kg olanzapine, mean plasma levels achieved of the anti-psychotic drug were ~178 ng/mL. The relatively high levels of olanzapine may help explain in part the hypophagic and adipsic phenomena experienced by rats at this particular dosing. Interpreted in this way, olanzapine concentrations ≥80 ng/mL (as in our studies) reached a threshold for the onset of malaise or taste aversion effects. In contrast, animals exposed to a 0.5 mg/kg olanzapine dose showed optimal therapeutic range of olanzapine plasma levels (~47 ng/mL) and normal feeding and drinking behaviors. It should be noted that the dosing paradigm implemented in our current studies yielded fluoxetine, norfluoxetine and olanzapine plasma concentrations similar to those reported by Zhang et al [4] under an acute experimental design. Therefore it is possible that little or no significant metabolic interactions between fluoxetine and olanzapine combination treatment occurs in rats as a function of chronic drug exposure. This possibility has merit as no clinically significant metabolic interactions are also reported during combined fluoxetine and olanzapine therapy [29]. Placing the current data in the framework of the growing body of experimental and clinical evidence, it is unlikely that drug-drug interactions modify the pharmacological profile of fluoxetine and olanzapine when the two psychoactive agents are administered concomitantly to experimental animal models.

## Conclusions

Combination therapies of anti-depressant and anti-psychotic drugs are increasingly used for treatment-resistant mood disorders. Here, we have provided further evidence that fluoxetine and olanzapine have pharmacodynamic event-related effects on body weight dynamics [5]. In rats, these effects are manifested in the form of anorexia or perhaps anhedonia to food and water. Of interest, anorectic phenomena are also observed in rats chronically treated with valproic acid and lithium [31]; both valproic acid and lithium are widely touted as effective prophylactic agents for manic-depressive illness [32]. It is quite probable therefore that augmentation therapy of several mood stabilizers is associated with weight *loss *in rats, whereas the same combination drug pattern results in weight *gain *in special patient populations. This disparity adds a new level of complexity to the issue of body weight changes associated with psychopharmacology [19], and indicates species-specific variations in this phenomenon. In our particular case, adjusting olanzapine dosing to rat studies from 5 mg/kg to 0.5 mg/kg should preclude malaise bouts and/or taste aversion effects. In addition, the above dosage modification should be considered for achieving clinically therapeutic anti-psychotic plasma levels. If such a dosing paradigm is overlooked, it may lead to erroneous conclusions regarding mechanisms of medication action and side effect profile during drug augmentation therapy.

## Methods

### Animals and drug administration

Adult male Long-Evans rats (Harlan, Indianapolis, IN) were used in all experiments described herein. Prior to any drug treatment, all rats were handled for 5 days to minimize non-specific stress. Rats were then randomly assigned to the various experimental groups and cage mates received the same drug treatment. Animals were group-housed, 2–3 per cage under a 12 hr light:dark cycle (light on 0700) and allowed *ad libitum *access to food and water, except when noted (see below). For the chronic drug regimen, rats were injected intraperitoneally (IP) first with fluoxetine (10 mg/kg) followed 15 min later by olanzapine (5 or 0.5 mg/kg) to decrease potential pharmacokinetic interactions. Fluoxetine was dissolved in 5% γ-cyclodextrin, whereas olanzapine was dissolved in 12% γ-cyclodextrin (cyclodextrin was used to improve the stability and bioavailability of poorly soluble drugs). Dosages of the two drugs were chosen according to each drug's *in vivo *potencies for affecting 5-HT, DA, ACh and H systems [15,33,34], and also from pharmacological doses reported in the literature [4,8,11,35,36]. Doses of drugs are expressed as their respective salts. Control animals received 5% and 12% γ-cyclodextrin injections (1 ml/kg) at 15 min intervals so that this group was given the vehicle-solution at the same times as the fluoxetine plus olanzapine experimental group. All injections were administered between 1000 and 1100 hr of the light cycle. All aspects of the following experiments were carried out in accordance with the NIH *Guide for the Care and Use of Laboratory Animals *and with approval from the NYIT IACUC.

### Experimental procedures

Rats were injected with fluoxetine plus olanzapine or their respective vehicle-solutions for 18 consecutive days and body weights recorded before (day 0) and after (day 18) drug treatment. In addition, body weights were also recorded 7 and 14 days after drug treatment to adjust for dosage. To determine average food intake over a 12 hr period, control-vehicle and experimental rats were given pre-measured food pellets (25 g/rat) on day 10 and subsequent consumption was recorded on the next day. To keep track of food spillage, cage bedding was randomly separated to assess degree of unconsumed food. A similar procedure was instituted to assess average water intake over a 12 hr period: both groups were given pre-measured tap water (100 mL/rat) on day 12 and subsequent consumption was recorded on the next day. On the last day of injections (day 18), rats were either decapitated or perfused under deep chemical anesthesia (ketamine/xylazine/acepromazine, 60 mg/kg) with 4% paraformaldehyde. Trunk blood or blood collected from cardiac puncture was collected in centrifuge tubes containing either no EDTA or a 0.3 M EDTA (pH 7.4) solution. Blood samples with 0.3 M EDTA were centrifuged at 10,000 RPM for 10 min and the collected serum frozen at -80°C until determination of glucose levels. Blood collected without EDTA was also frozen at -80°C until determination of drug plasma concentrations by GC-MS.

### Glucose measurements

To determine relative glucose levels, all animals were fasted for 12 hr on the last day of injections (i.e., day 18). Glucose serum levels were determined using the Life-Scan One Touch Basic Meter (Johnson & Johnson, New Brunswick, NJ). In brief, 10 μl of serum was pipetted as a free-flowing drop onto each One Touch Test Strip and read in the glucose meter for 45 sec. Fasting glucose levels are presented as means ± SEM in mg/dL.

### Analysis for fluoxetine, norfluoxetine and olanzapine by GC-MS

All solvents used for drug analyses were HPLC grade. Chloroform and 1-chlorobutane were obtained from Burdick & Jackson (Muskegon, MI). Ethyl Acetate and acetonitrile were obtained from EMD (Gibbstown, NJ). Ammonium Hydroxide, ACS certified, was obtained from Fisher (Fairlawn, NJ). Ascorbic acid was obtained from Sigma (St. Louis, MO), whereas trifluoroacetic anhydride was obtained from Pierce (Rockford, IL).

Working solutions containing olanzapine, fluoxetine and norfluoxetine at 10 ng/μL, 1.0 ng/μL, and 0.1 ng/μL, respectively were prepared in methanol. The working solutions used to prepare the calibration standards and controls were derived from different sources of reference material (e.g., fluoxetine) or different weighing of the same reference material (e.g., olanzapine). Calibration standards and controls were prepared by adding appropriate amounts of working solutions to clean, separate silanized 16 × 100 mm culture tubes that contained 1 mL of blank bovine blood and 0.2 mL of 2.5% ascorbic acid. The calibration standards ranged from 1 ng/mL to 1000 ng/mL. The controls were prepared at 35 ng/mL, 100 ng/mL, and 650 ng/mL.

A 0.25 mL volume of each blood sample was transferred to clean silanized 16 × 100 mm culture tubes. To bring the sample preparation to a volume of 1 mL, a 0.75 mL volume of Milli Q H_2_O was added to each tube. The samples were therefore a 4-fold dilution compared with the standards and controls. A 0.2 mL volume of 2.5% ascorbic acid was added to each sample in a preparation tube. Sample, standards, and controls were extracted by a liquid/liquid procedure. Eighty ng of fluoxetine-d_6 _(80 μL of 1 ng/μL fluoxetine-d_6 _in Milli Q H_2_O) and 80 ng of clozapine (80 μL of 1 ng/μL clozapine in methanol) were added to each tube and the tubes were then briefly vortexed. A 0.1 mL volume of the concentrated ammonium hydroxide and a 4 mL volume of 1-chlorobutane: acetonitrile (4:1) was then added to each tube. A clean teflon-lined screw cap was placed on each tube. The tubes were mixed 20 min using a reciprocating shaker and centrifuged at 2000 rpm for 10 min using an IEC centrifuge (Needham, MA). Using clean, separate glass Pasteur pipettes, the upper organic layer from each tube was transferred to clean, separate 13 × 100 mm culture tubes. The organic layer was evaporated to dryness under a stream of air at 40°C using a Turbo Vap evaporator (Zymark Corporation, Hopkinton, MA).

For derivatization, a 0.1 mL volume of chloroform and a 0.1 mL volume of trifluoroacetic anhydride were added to each tube. Clean teflon-lined screw caps were then placed on each tube. The tubes were heated for 20 min at 70°C using a dry block heater. After heating, the tubes were removed from the heater and allowed to cool at room temperature. The caps were then removed and the tubes evaporated using the same conditions as described above. Derivatized extracts were reconstituted with 100 μL of ethyl acetate and were then transferred to clean, separate auto-sampler vials.

The GC-MS system consisted of an Agilent 6890 gas chromatograph and an Agilent 5973 MSD mass spectrometer (Palo Alto, CA). The data system consisted of a Hewlett-Packard X A 6/400 computer and Agilent Chemstation software. For chromatographic separation, a ZB-5, 30 meter × 0.25 mm id, 0.25 μm capillary column (Phenomenex, Torrance, CA) was used. The carrier gas was ultra high purity helium at a flow rate of 1.0 mL/min. The injection port temperature was 260°C and the transfer line temperature was 300°C. The column oven temperature program was 125°C, held at this temperature for 0.2 min, and then increased to 300°C. Positive chemical ionization was used for the mass spectrometry analysis. The ion source temperature was 200°C and ammonia was used as a reagent gas. Selected ion monitoring was used and the following ions (m/z) were analyzed: norfluoxetine: 409, fluoxetine: 423, fluoxetine-d_6_: 429, olanzapine: 409, clozapine: 423. For fluoxetine, norfluoxetine, and fluoxetine-d_6_, the protonated ammonia adducts of the molecule ions were monitored. For olanzapine and clozapine, the protonated molecule ions were also monitored. Fluoxetine-d_6 _was used as the internal standard for fluoxetine and norfluoxetine. Clozapine was used as the internal standard for olanzapine. The limit of quantification for fluoxetine, norfluoxetine and olanzapine was 5 ng/mL. Data are presented as the means ± SEM.

### Data analysis

Statistical comparisons in body weight and glucose levels were carried out using one-way ANOVA or two-tailed *t *tests where appropriate. Plasma levels of fluoxetine and olanzapine were analyzed by a Student's *t*-test. The probability level interpreted as significant was P ≤ 0.05.

## Authors' contributions

JAP and JMC participated in the in vivo studies and in the biochemical assays. BHH and JMH participated in the design of the studies and performed the statistical analysis. GT drafted the manuscript, conceived the study and participated in its design and coordination. All authors read and approved the final manuscript.
